# Towards a standard protocol for antimony intralesional infiltration technique for cutaneous leishmaniasis treatment

**DOI:** 10.1590/0074-027601700125

**Published:** 2018-02

**Authors:** Rosiana Estéfane da Silva, Janaína de Pina Carvalho, Dario Brock Ramalho, Maria Camilo Ribeiro De Senna, Hugo Silva Assis Moreira, Ana Rabello, Erika Cota, Gláucia Cota

**Affiliations:** 1 Fundação Oswaldo Cruz-Fiocruz, Centro de Pesquisas René Rachou, Centro de Referência em Leishmanioses, Pesquisa Clínica e Políticas Públicas em Doenças Infecto-Parasitárias, Belo Horizonte, MG, Brasil; 2 Universidade Federal do Rio Grande do Sul, Instituto de Informática, Porto Alegre, RS, Brasil

**Keywords:** cutaneous leishmaniasis, therapy, meglumine antimoniate, intralesional infiltration, validation

## Abstract

**BACKGROUND:**

Despite its recognised toxicity, antimonial therapy continues to be the first-line drug for cutaneous leishmaniasis (CL) treatment. Intralesional administration of meglumine antimoniate (MA) represents an alternative that could reduce the systemic absorption of the drug and its side effects.

**OBJECTIVES:**

This study aims to validate the standard operational procedure (SOP) for the intralesional infiltration of MA for CL therapy as the first step before the assessment of efficacy and safety related to the procedure.

**METHODS:**

The SOP was created based on 21 trials retrieved from the literature, direct monitoring of the procedure and consultation with experts. This script was submitted to a formal computer-aided inspection to identify readability, clarity, omission, redundancy and unnecessary information (content validation). For criterion and construct validations, the influence of critical condition changes (compliance with the instructions and professional experience) on outcome conformity (saturation status achievement), tolerability (pain referred) and safety (bleeding) were assessed.

**FINDINGS:**

The median procedure length was 12 minutes and in 72% of them, patients classified the pain as mild. The bleeding was also classified as mild in 96.6% of the procedures. Full compliance with the SOP was observed in 66% of infiltrations. Despite this, in 100% of the inspected procedures, lesion saturation was observed at the end of infiltration, which means that it tolerates some degree of modification in its execution (robustness) without prejudice to the result.

**CONCLUSIONS:**

The procedure is reproducible and can be used by professionals without previous training with high success and safety rates.

Cutaneous leishmaniasis (CL) is an endemic zoonosis transmitted by the bite of infected sandflies that causes lesions on exposed parts of the body and that has the potential to leave scars for life. Approximately two-thirds of all cases are concentrated in six countries: Afghanistan, Algeria, Brazil, Colombia, the Islamic Republic of Iran and the Arab Republic of Syria and, according to World Health Organization records, the number of CL cases tripled in high-burden countries from 1998 to 2014 (from > 50,000 in 1998 to approximately 150,000 in 2014) (WHO 2016). In total, cutaneous and visceral leishmaniasis are responsible for more than 3,300,000 days lost or days where some disability was experienced disability - adjusted life years (DALY) (Murray et al. 2012).

Despite its recognised toxicity, antimonial therapy has been employed for several decades and continues to be the first-line drug for CL treatment in many countries (WHO 2010). Intralesional administration of meglumine antimoniate (MA) represents an alternative that could reduce the systemic absorption of the drug and its side effects. Although widely used in the Old World, in the Americas, the experience with this therapeutic modality is still limited (Oliveira-Neto et al. 1997, Soto et al. 2013). The accumulated experience with the intralesional approach in Brazil suggests the efficacy and safety of the procedure. However, there are few detailed descriptions available for the intralesional technique, and there are several procedure variations, which makes it difficult to compare results, and thus, the implementation of this technique on a large scale is limited. In the past decade, the evidence basis for how processes of care affect outcomes have solidified, evolving in parallel with the concept of evidence-based medicine. In this context, the incorporation of this new therapeutic approach first requires its validation as a standardised process. The present study aims to develop and validate a tool to guide the MA intralesional infiltration procedure for CL treatment, a standard operational procedure (SOP). The process of developing and validating a set of instructions that guides the execution of the infiltration procedure will be the focus of this paper, which will not present data on the efficacy of the treatment itself, since it would require the analysis of other clinical parameters that are beyond the scope of this study.

## SUBJECTS AND METHODS

The intralesional infiltration SOP was developed following a comprehensive and progressive process, and the ordered pool of instructions (script) built to guide the procedure was submitted to a series of rigorous tests of reliability based on the analyses of content, criterion and construct. Considering the intralesional infiltration a medical procedure and, as such, an action encompassing both understanding and knowledge, to validate the SOP, we chose to apply the psychometric principles (Beard et al. 2011) associated with automated methods of software inspection (Ribeiro et al. 2014).

*Study phase I: the development of the SOP* - As the first step, a systematic search of all descriptions of the procedure available in the MEDLINE and LILACS databases was performed in July 2015 using a sensitive strategy combined by Boolean operators. There was no language restriction, but the research was limited to the last 10 years, and the filter “clinical trial” was used. Additional studies were located by a manual search using references from retrieved papers, and trial register databases were also consulted. All identified studies were read in full focusing on the description of the intralesional procedure, specifically: anesthesia use; needle gauge specification, tilt, direction and depth during the infiltration; drug volume infiltrated or any criteria used for stopping the infiltration.

**Fig. 1:**
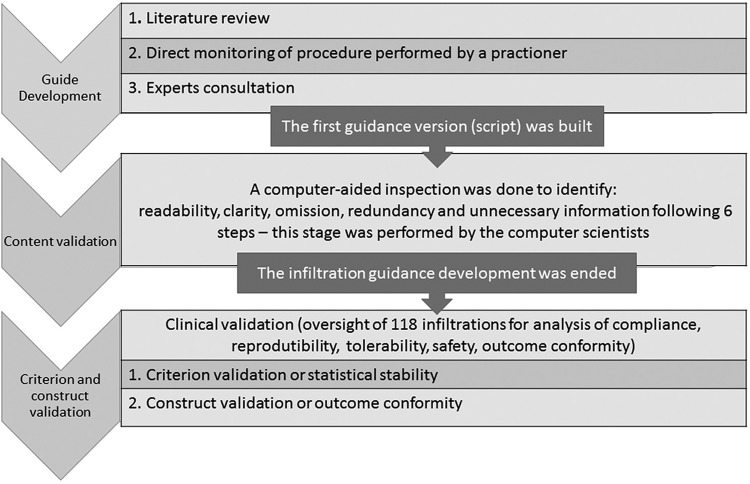
flowchart of standard operational procedure development process.

In addition, to identify all the stages comprising the intralesional infiltration process, an observation of the procedure being performed by an experienced professional in a specialised CL centre was also performed. However, even with these attempts (theoretical and practice) of gathering and sorting all actions necessary to carry out the infiltration, it was observed that there were still many conflicting or unresolved questions in the set of instructions. So, a panel of expert investigators with wide experience in the infiltration technique was consulted on the unsolved issues. All principal investigators in those published studies were identified and were contacted electronically and invited to answer a multiplechoice questionnaire. The investigators could also make any comment that they judged necessary. In the case of more than one publication from the same group, contact was made with the group coordinator, often identified as the last author on the manuscripts. Consultation with experts ended the first phase of this work, which resulted in the development of the first intralesional infiltration SOP version (script). An overview of the development and validation process is shown in a flowchart in Fig. 1.

*Study phase II: validation of the first SOP version (script)* - The reliability of the intralesional infiltration SOP was measured using the generalisability psychometric theory in a second study phase, where the content, criterion and construct aspects were validated (Beard et al. 2011, Raza et al. 2015). In other words, the assessment of reliability included the following aspects: (i) the internal consistency of the script (content validation); (ii) its stability to changes in critical conditions (criterion validation); (iii) the measurement of the extent to which the SOP leads to the intended result (construct validation).

The content validation of the intralesional infiltration script was performed systematically by a specific software product and is detailed in the Content Validation section. This computer-aided process identified several issues in the set of instructions, which were translated into queries by computer scientists and answered by the medical team. This interactive process generated after several rounds a reviewed and improved version of the script, now renamed SOP, which was implemented as the operational procedure to be followed during an ongoing clinical study addressing the efficacy and safety of AM intralesional therapy. The clinical study was submitted and approved by the Ethical Review Board of Centro de Pesquisas René Rachou (CPqRR), FIOCRUZ (approval number 1.136.132). In addition, the study protocol was registered on the Brazilian Clinical Studies Registry Database (REBEC) under number RBR-44KG5X.

A total of 17 medical doctors participated in the study. Initially, only three professionals with previous experience in intralesional infiltration (medical doctor seniors - permanent members of the clinic staff) performed the procedure. After six months, 14 training doctors (temporary staff: one month infectious diseases training residents) could also run the infiltration after reading the SOP. For criterion and construct validation, all the intralesional infiltration procedures were inspected by a single observer who filled out a checklist containing questions about the professional's adherence to the SOP and other features of the procedure, including a numeric visual scale of pain suffered by the patient.

*Content validation* - Content validation refers to the judgment of the quality of the description of the tool (script) or, in other words, if it covers all the actions included in the proposed procedure (intralesional infiltration) and does not contain elements (or actions) related to other procedures. A formal content evaluation method (Ribeiro et al. 2014) - a previously validated tool for verification of software specification documents - was applied (Cota et al. 2017). This method was developed by computer scientists from Federal University of Rio Grande do Sul, Brazil, and it is based on six steps:

*Step 1: Pre-processing of the original script* - This step consists of translating the first SOP version (script) to a computer notation where each action corresponds to one instruction in the procedure.

*Step 2: Identification of entities* - Consists of identifying the entities that appear in the script. Usually, the entities in a document are represented by nouns or noun phrases, such as “*medical doctor”*, *“2% lidocaine”*, *“syringe”*.

*Step 3: Identification of actions* - The next step is to identify the essential actions to be performed separately. Actions are usually represented by verbs and verb phrases and correspond to operations involving the entities. The first step of the methodology makes this step simpler as one can extract one action for each translated instruction.

*Step 4: Characterisation of conditions and effects as states* - This is the modeling step, where the script written in natural language (medical terms) is described using mathematical notation. After extracting the entities and actions presented in the script, graphical representations for each one of them were defined to facilitate the visualisation of the mathematical model. Each part of the graphic is an element of a graph transformation (GT) model, i.e., a mathematical structure composed of nodes and arcs with an associated semantic that will be the target of further analysis steps.

*Step 5: Construction of rules* - This step consists of creating transition rules for each action listed in Step 3. A rule for an action is modeled as a transition between two states modeled in Step 4.

*Step 6: Analysis* - With all those artifacts built in the previous steps, several automatic analyses are performed to detect problems with the script. The “conflict analysis”, “dependency analysis” and “rule sequence analysis” can raise open issues (OIs) regarding either some aspect of the instructions or the modeling.

*Criterion validation* - Criterion validation indicates the degree to which changes in process/performer influence the outcome; in this case, the outcomes were saturation status achievement and tolerability (scale of pain referred and bleeding score) and the critical conditions are the lack of adherence to the instructions provided in the SOP and professional experience. The statistical stability (robustness) of the tool was established by determining the inter-rater (comparison between the professionals) and test-retest (consistency of scoring over time) reliabilities. A structured questionnaire (checklist) with ten questions covering the essential actions and concepts (domains) considered essential to the achievement of the planned outcome (Supplementary data, Figure) was developed. The checklist was filled in by only one observer (study investigator) to identify whether a planned action was executed, if it was executed correctly, incorrectly or semi-correctly and if it was carried out in the sequence described. During the inspection of infiltrations, the study investigator asked the executor of the procedure (medical doctor) to inform which criterion he/she used to suspend the infiltration. When the executor finished the procedure, the study investigator evaluated whether the saturation state had been reached according to the previously adopted criterion: volume increase of the entire lesion, with or without pallor. Thus, with the checklist, a score from 0 to 10 indicating the degree of adherence to the instructions provided in the SOP was obtained. For assessment of the pain referred by the patient during the procedure, the visual analogue scale (VAS) (Gift 1989), an illustrated and continuum scale comprised of a horizontal line ranging from none to an extreme amount of pain defined as 10, was applied. For the bleeding assessment, the Fromme-Boezaart modified scale (Boezaart et al. 1995), a surgical field bleeding scale from 0 to 5 that is in accordance with blood cleaning requirements, was used. This scale was proposed for evaluation of surgical sites using the suctioning requirement as the bleeding intensity measurement. Suctioning is a kind of procedure not performed in outpatient setting, scenario of the intralesional infiltration. Even so, the score is easy to be applied and has been modified, being “suctioning” replaced by “bleeding removed by gauze”.

*Construct validation* - The aim is to demonstrate that the process produces the planned result, namely, that the result obtained conforms to predetermined “target”. The variables chosen for construct validation included the saturation status achievement at the end of infiltration, following the definition established by the consultation of experts; the tolerability of the procedure, as evaluated by the visual analogue scale of pain; and safety, as evaluated by the bleeding score.

*Statistical analysis* - The sample (number of infiltrations to be observed in reliability assessment) was determined considering a confidence level of 95% (Z = 1.96) and a maximum error margin of 20% for all domains of interest (dichotomous data). Based on that, a sample of 24 procedures was estimated for each group for all comparisons necessary for the evaluation of statistical stability (experienced versus not experienced professionals, same professional's group over time), and the minimum number of intralesional infiltrations requested for the procedure validation was calculated as 72. Descriptive statistics were calculated for different groups in each domain. Statistical analysis was conducted using the Student's *t*-test, analysis of variance, the Wilcoxon signed-rank test (for nonparametric variables), chi-square, McNemar test (two related dichotomous variables) and One-Way ANOVA, whenever appropriate. The Spearman correlation coefficient and the 95% confidence interval (95% CI) for the correlation coefficient was used to compare the professional's adherence to the SOP according to their experience status. A logistic regression analysis model (using backward stepwise) for bleeding intensity was built adjusting covariates identified by univariate analyses with a p < 0.2. Statistical significance was set at the 0.05 level. All analyses were performed using Statistical Product and Service Solutions, IBM - SPSS® (version 23, California, USA).

## RESULTS

Through literature review, 19 (Firooz et al. 2005, Shazad et al. 2005, Negi et al. 2007, Munir et al. 2008, Layegh et al. 2009, El-Sayed & Anwar 2010, Kashani et al. 2010, Van Thiel et al. 2010, Meymandi et al. 2011, Safi et al. 2012, Bumb et al. 2013, Khatami et al. 2013, Mohammedzadh et al. 2013, Soto et al. 2013, Dieterle & Pillekamp 2014, Stahl et al. 2014, Banihashemi et al 2015, Parizi et al. 2015, Ranawaka et al. 2015) clinical trials addressing intralesional therapy over the last 10 years were identified. Two additional studies, references from primary articles, were also included in the analysis (Oliveira-Neto et al. 1997, Zeglin 2009), totalising 21 studies reviewed.

The studies were performed between 2005-2015, mostly in the Old World, namely, in Iran (9), Afghanistan (5) India, (2) Sir Lanka (1), Pakistan (1), Egypt (1), Bolivia (1), and Brazil (1). The intralesional infiltration technique was summarily described in almost all studies, limited to the interval between the infiltrations and the maximum number of applications. In the two publications from the Americas (Oliveira-Neto et al. 1997, Soto et al. 2013) anesthesia with lidocaine before the infiltration was recommend, unlike in the old-world studies. Although the expression “intradermal infiltration” was almost always used, only in one study (Shazad et al. 2005) the depth of the infiltration was detailed as “in the upper and mid dermis”. Except for one author (Soto et al. 2013), who advocates the use of a fine-gauge needle (less than 25 gauge), all the others who mentioned the needle caliber (Oliveira-Neto et al. 1997, Firooz et al. 2005, Shazad et al. 2005, Khatami et al. 2013) have recommended needles between 30 and 40 gauge. No author mentioned the needle tilt; however, concerning the needle direction, four studies (Firooz et al. 2005, Khatami et al. 2013, Soto et al. 2013), described the procedure starting from the periphery to the centre of the lesion, moving the needle in all directions. Some authors recommended different volumes or doses of drug based on the area of the lesion (Firooz et al. 2010, Soto et al. 2013, Bumb et al. 2013), while others (Oliveira-Neto et al. 1997, Shazad et al. 2005, Khatami et al. 2013) proposed that the application should be of all the volume required to cause a visible change in the lesion (“saturation” or “blanching”), an aspect that is described as the desired outcome after infiltration in all studies. In turn, the direct monitoring of the infiltration procedure performed by a practitioner showed that the drug was injected only at the edges of the lesion. Thus, the following issues were considered unsolved:

1. Should the intralesional approach be indicated for non-ulcerated lesions?
2. How should the needle be directed during infiltration?
3. What criteria must be observed for the definition of lesion saturation?

These remained conflicting or missing issues were submitted electronically to a panel of experts through objective questions - a methodology based on Delphi technique (Hsu & Sandford 2007). The approach includes several rounds of discussion to reach consensus. The experts were identified from the 10 research groups identified during the literature review. All of them were contacted for the resolution of unsolved issues, and only five of them responded to the questionnaire. All five experts considered intralesional therapy applicable to non-ulcerated lesions. Additionally, unanimously, all of them defined “saturation” as the presence of swelling. Three investigators indicated “elevation of the base of the lesion” as an observable manifestation for the understanding of the establishment of saturation, while two others defined saturation as “a disappearance of the gap between the centre and edge of the lesion”. Only one consulted investigator considered the presence of pallor as an indispensable condition for achieving the saturation state. Based on that, the technique adopted was “to insert the needle toward the centre of the lesion and to move it back toward the edge while gently and continuously infiltrating the medication. The same movement is repeated in the radial direction until reaching the saturation of the full lesion: infiltration from each insertion point is directed in a V-shaped pattern towards the centre and the margin, together covering the bed of the entire lesion”.

Finally, some definitions followed the usual understanding in elective skin surgical procedures (BAD 2014) namely, local cleaning with antiseptic solution prior to the procedure and buttons of anesthetics in the cardinal points adjacent to the lesion, preferably carried out with sterile gloves. Based on the knowledge of the skin layers, the recommendation to keep the needle in a parallel plane to the base of the lesion and with the bevel facing up to fill the dermis layer affected was also included in the SOP. So, it is possible to assume that the infiltration occurs deeply in the dermis and possibly in the adjunctive subcutaneous tissue. Considering the lack of available pharmacokinetic data for antimony administered by intradermal route, we opted for a more conservative approach and limited the maximum daily dose to that recommended for parenteral use (three ampoules), according to the current recommendations (OPAS 2013). As in other skin procedures, the decision to use or not use a vasoconstrictor in association with lidocaine was left to the best medical understanding of the lesion site and the patient's characteristics. For the choice of the needle gauge we have adopted that one already used successfully in our service (25G x 0.7mm) into thread connection syringes. In the execution of the clinical study that supported the validation of intralesional infiltration SOP, the current recommendations for conducting CL clinical trials were followed, as proposed by Olliaro and collaborators in 2013, including the formula for calculating the area of the lesion, the definitions for the outcomes of interest and its moment of evaluation. Specifically, the lesion area was defined as the product of the two largest internal diameters of the ulcer (Olliaro et al. 2013).

The first step of validation, the content inspection, raised several ambiguities present in the first SOP version (script). As an example, the word “medication” was used to refer to both, 2% lidocaine and Glucantime®. Although a specialist can infer which exact medication was meant in each statement of the SOP, the word could cause misunderstandings in less attentive or less experienced physicians. The model analysis raised a question about possible different scenarios, such as different ulcer sizes and shapes. Even though a physician is supposed to identify and adjust the procedure accordingly, leaving these scenarios out of the scope of the SOP results in a variability in the application of the procedure, which goes against the proposed standardisation, so this too was modified in the following version.

**Fig. 2:**
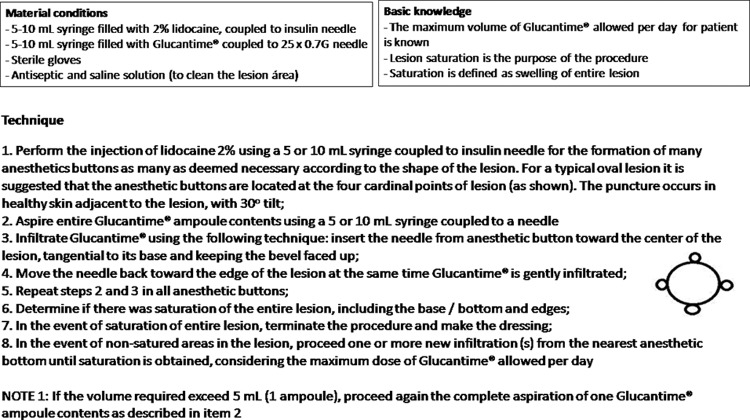
final intralesional infiltration standard operational procedure (SOP) version.

Finally, the script was transformed into a more detailed tool encompassing a wider range of either small lesions, which require only an anesthetic point, or larger lesions requiring many anesthetic points to reach the entire extension of the lesion. In addition to that, an additional infiltration step, in the case of saturation not being achieved, was included in the script. This modified script was re-modeled and re-verified, which generated a few additional small issues. Thus, after four verification rounds, a more detailed SOP covering many possible scenarios that can be faced by the physician during the intervention was achieved (Fig. 2).

A total of 118 infiltrations were carried out over a 12-month period (August 2015 to July 2016) in 38 patients (the average of procedures per patient was three) after signing the consent form. Eighty-nine infiltrations were performed by three professionals with some previous experience in intralesional technique and 29 procedures were done by 14 physicians in training. The median total area of the lesions was 429 mm^2^ and the median volume of Glucantime® administered on the first day of treatment was 5 mL (25-75% interquartile range 2.3 to 8mL).

**Fig. 3:**
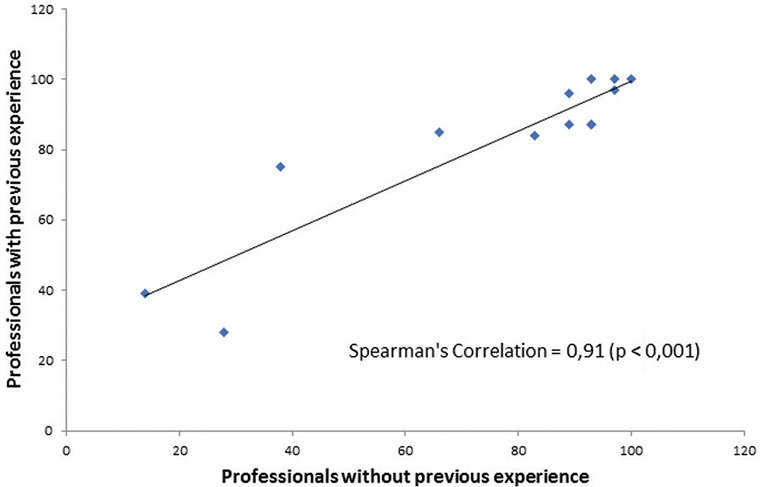
the Spearman correlation coefficient for professional's adherence to the standard operational procedure (SOP).

The median procedure length was 12 min (ranging from 3 to 35 min), and in 72% of them, patients classified the pain during infiltration as mild (up to 3 in a visual analogue scale that goes from 0 to 10). The bleeding was also classified as mild (less than or equal to 3 on the modified Boezaart scale) in 96.6% of the procedures. Considering the items assessed by the checklist, full compliance with the SOP was observed in 66% of infiltrations. Despite this, in 100% of the inspected procedures, lesion saturation was observed at the end of infiltration. Instructions with a lower compliance rate were a needle tilt non-parallel to the base of the lesion during infiltration (20% of procedures), incomplete aspiration of the ampoule contents before starting the procedure (16% of procedures) and insertion of the needle into the lesion and not on intact skin (13% of procedures). Failure to comply with anesthesia procedures occurred in 12% of infiltrations. In all observed procedures, the recommended maximum limit of medication per day was observed. The professional's adherence to the SOP according to the previous experience was compared by univariate analysis to test the statistically stability (Table I). The Spearman correlation coefficient for professional's adherence to the SOP (experienced and not experienced medical doctors) is presented in Fig. 3. In this Figure, each point represents one of the criteria observed during the inspection of the procedures - detailed in Supplementary data, Figure (checklist) - and demonstrates the monotonic relationship between the paired data, in this case, the performance of the two groups of professionals, according to their previous experience. The parameters that differed between experienced and non-experienced medical doctors during the infiltration procedure execution were “*full adhesion to the SOP*”, “*needle tilt parallel to the base of the lesion*” and “*bleeding scoring*”. The last observation, the highest proportion of patients reporting bleeding with intensity greater than 3 during procedures performed by experienced medical doctors, was an unexpected result and required a logistic regression analysis to be analysed. All variables related to bleeding with a p < 0.2 in univariate analyses, namely “*needle insertion from intact skin*” (p = 0.14), *“needle tilt parallel to the base of the lesion”* (p = 0.12), “lesion area” (p = 0.01) were included in the initial model. The only factor related to bleeding intensity was the cutaneous lesion size (odds ratio 1.004, 95% confidence interval 1.000-1.004, p = 0.01).

**TABLE I:** Results of the procedure inspection according to professional experience

	Professionals without prior experience in therapy IL (Number of procedures: 29; number of professionals: 14)	Professionals with previous experience in therapy IL (Number of procedures: 89; number of professionals: 3)	p value
Time spent with the procedure in minutes (median, min-max)	13.5 (4-21)	12 (3-35)	0.21**
100% adhesion to the SOP	11/29 (38%)	67/89 (75%)	0.00*
Pain VAS scoring above 3	8/29 (28%)	25/89 (28%)	1*
Bleeding FBS scoring above 3	4/29 (14%)	35/89 (39%)	0,01*
Local anesthesia administration before infiltration	27/29 (93%)	77/89 (87%)	0.51*
Needle insertion from intact skin	26/29 (89%)	77/89 (87%)	1*
Complete ampoule contents aspiration before starting the infiltration	24/29 (83%)	75/89 (84%)	1*
Needle insertion from anesthetic button	27/29 (93%)	77/89 (87%)	0.51*
Needle position toward the center of the lesion	28/29 (97%)	85/89 (97%)	1*
Needle tilt parallel to the base of the lesion	18/29 (66%)	76/89 (85%)	0.02*
Placement of the bevel of the needle facing up	27/29 (97%)	89/89 (100%)	0.15*
Slow drug infiltration while rewinding the needle	26/29 (89%)	85/89 (96%)	0.36*
Understanding of the concept of saturation as swelling of the lesion	27/29 (93%)	89/89 (100%)	0.06*
Maximum drug limit per day was observed	29/29 (100%)	89/89 (100%)	-

FBS: modified Fromme-Boezaart scale; IL: intralesional; SOP: standard operational procedure; VAS: visual analogue scale;

* p value is computed using chi-square test;

** p value is computed using one-way ANOVA test.

Considering that there is no consensus on the requirement to perform local anesthesia prior to intralesional infiltration, we evaluated whether the intensity of pain reported by the patients was related to violation of the anesthesia procedures recommended in SOP (Table II). In the univariate analysis, only “*infiltration from nonintact skin*” was associated with the intensity of pain reported by the patients (p = 0.03).

**TABLE II:** The anesthetic recommendations adhesion during intralesional infiltrations grouped according to pain intensity referred by patients

	Pain VAS scoring until 3 (referred after 86 procedures)	Pain VAS scoring above 3 (referred after 32 procedures)	p value*
Local anesthesia administration not performed before infiltration	7/86 (8%)	7/32 (28%)	0.06
Needle insertion not performed from intact skin	7/86 (8%)	8/32 (25%)	0.03
Needle insertion not performed from anesthetic button	7/86 (8%)	7/32 (22%)	0.06

VAS: visual analogue scale;

* p value is computed using chi-square test.

To assess the changes in the outcomes of interest over time (Table III), we compared the first 45 infiltrations carried out during the validation study period performed by senior medical doctors (permanent staff) with the last 44 infiltrations carried out by these same professionals using statistical tests for paired samples. The percentage of compliance with the instructions increased and the score of pain reported by the patients reduced significantly over six months.

**TABLE III:** Results of procedure inspection over time (procedures performed by professionals with previous experience with intralesional infiltration)

	The first 44 infiltrations performed by medical doctor seniors	The latest 45 infiltrations performed by medical doctor seniors	p value*
Time spent with the procedure in minutes (median, min-max)	11 (3-27)	12 (4-35)	0.11
Complete adhesion to the SOP	26/44 (59%)	41/45 (91%)	0.00
Pain referred by patient above 3 or mild pain	19/44 (43%)	6/45 (13%)	0.01
Bleeding FBS scoring above 3	22/44(50%)	13/45 (29%)	0.22

FBS: modified Fromme-Boezaart scale; SOP: standard operational procedure;

* p value is computed using McNemar test.

## DISCUSSION

Evidence of validity and reliability are essential characteristics of evidence-based medicine, and the main consideration of a well-designed evaluation system is to ensure that the evaluation methods adopted are valid and reliable.

According to the 32nd Report produced by the WHO (WHO 1992), validation is “the documented act of proving that any procedure, process, equipment, material, operation or system really leads to the expected results”. There are several experiences in validating tools developed to assess psychomotor skills of medical trainees, allowing evaluation of competence (Beard et al. 2011, Raza et al. 2015). Although similar, in our case, the challenge was to validate a technical document with an instructive function (different from a “learning assessment method”) and a medical process. As additional difficulties, the result of the process being evaluated is an outcome determined by multiple factors and therefore a result not easily measured. Besides that, a unique technique description for the process is not available; instead, significant differences are observed between the approaches undertaken by the various practitioners.

The content validation is an important step toward treatment guide standardisation, but it is still a manual and *ad hoc* process in the medical community and decisions concerning conflicting actions are taken based on intuitive aspects identified by the observers, based on prior knowledge and individual experience with the issue at hand. As in any document written in natural language, procedure guides are prone to errors and misunderstandings caused by ambiguities, omissions, or other inconsistencies that are difficult to detect in an informal revision. In addition, fault detection in the field incurs additional costs and re-work. Our results show that a formal SOP verification can pinpoint several issues early that would otherwise remain unknown and might compromise the applicability of this health tool. Specifically, *conflict analysis* tells us which actions in the SOP represent points of decision, where conditions and/or responses to the actions must be checked. By running this analysis, one is forced to check whether all possible alternatives/scenarios of the medical procedure are described or can be achieved somehow by following the script. The formal inspection guided by the computer also systematically identifies relationships between rules. This *dependency analysis* is used to check whether the dependencies that are intuitively expected to occur are explicitly described. Finally, during the *rule sequence* analysis, it is possible to check whether the overall effect is the desired one and whether all possible outcomes are clearly considered and described in the script. Other issues raised are related to omission of information. In most cases, errors of omission occur because a certain tacit or technical knowledge is assumed in the document. Details of conflict, dependency and rule sequence analysis can be found in Cota et al. (2017).

For criterion validation, what we aimed was to assess the degree of consistency of outcomes between different performers and the SOP's adhesion rates. In the face of the lack of universal parameters that can be applied to all criterion validations, these parameters were anticipated as the main causes of variation in the intralesional infiltration procedure. Our analyses suggest that the proposed procedure is robust, which means that it tolerates some degree of modification in its execution, according to the professional, without prejudice to the result. At the other hand, the increase in the adhesion rate to the instructions of the SOP reveals that the quality of the work of the professionals can improve over time. Although it has been demonstrated for experienced professionals in this study, this could possibly occur with any professional. In addition, the intralesional infiltration procedure proved to be a reproducible technique usable by professionals without previous training with high success and safety rates.

The low rate of a full adhesion to the SOP's instructions was mainly due to problems related to needle tilt during infiltration. It reflects a mechanical difficulty among less experienced professionals (an inability to perform the procedure as described in SOP) depending on the location, depth and size of the lesion. One possible explanation would be the fact that most of the invasive procedures performed on the skin by doctors using a needle are performed with a 30-degree inclination, such as local anesthesia and vascular puncture. On the other hand, even without fulfilment of the needle tilt recommendation, our results demonstrate that 100% of the procedures reached the desired result, i.e., the saturation of the lesion. This could mean that the needle tilt determined in SOP, a recommendation based only on the rational theoretical (to fill the skin layers known to be afflicted by the disease), would not be a requirement for the procedure. However, the implication of this technical detail on the cure rate has not been evaluated yet. Any therapeutic procedure ultimately should produce a cure or at least a favorable response with remission of the lesion. However, in this case, cure is not an immediate expected result after the procedure; there are many factors determining CL cure, including variables related to the parasite and the host, regardless of technical correction in any route of administration of the drug. Thus, by consulting experts, it was defined that the desired result of the intralesional infiltration procedure was the saturation of the lesion, defined as swelling of the entire lesion accompanied or not by pallor. Therefore, in addition to saturation achievement and considering the surgical nature of the approach, which could potentially produce pain, the patient's tolerance assessment system was also evaluated through a standardised pain scale, providing one more feasibility measure of the procedure. These findings suggest an association between pain intensity and some failure in the procedures recommended for local anesthesia.

The high percentage of cases achieving the desired outcome, the mild bleeding, the patients’ high tolerance and the reliability presented in this study confirm that the procedure is feasible and is fit to be tested in future clinical trials addressing effectiveness. In short, the SOP presented here was developed taking account not only the Brazilian experience (Oliveira-Neto et al. 1997, Vasconcellos et al. 2012, Duque et al. 2016, da Silva et al. 2016) but also all the relevant aspects of the technique identified in the main experiences with this therapeutic procedure already reported. The SOP represents a useful tool to guide the MA intralesional infiltration for CL treatment within the good clinical practice principals, ensuring uniformity. In addition, the SOP was evaluated by accepted methods to guarantee clarify and correction, and after that, validated in relation to its robustness and reproducibility. All these aspects make this standardisation a useful instrument for the effective incorporation of the antimoniate meglumine intralesional infiltration approach, as recently proposed by the Ministry of Health in Brazil (MS 2017).
