# Supplementary material for: Towards a standard protocol for antimony intralesional infiltration technique for cutaneous leishmaniasis treatment
Source: Mem Inst Oswaldo Cruz. 2018 Feb;113(2):71–9. doi: 10.1590/0074-027601700125 (PMC5722261; doi:10.1590/0074-027601700125)
Supplement: Supplementary file 1 [file 0074-0276-mioc-113-02-0071-Suppl01.pdf]

	Yes	No
1. To proceed lidocaine infiltration using an insulin needle		
2. The anesthetic button is made on intact skin (an area adjacent to the lesion)		
3. To aspirate completely the contents of Glucantime ampoule (5 ml)		
4. To introduce the needle from anesthetic button		
5. To introduce the needle towards the center of the lesion		
6. Needle inclination tend to be parallel to the base of the lesion		
7. To keep the bevel faced up		
8. To move the needle back toward the edge while gently infiltrating the medication		
9. To know saturation as swelling/edema (with or without paleness)		
10. To respect the maximum volume of Glucantime allowed		

	FROMME-BOEZAART MODIFIED SCALE
GRADE 0	No bleeding
GRADE 1	Slight bleeding. No bleeding removed by gauze is required.
GRADE 2	Slight bleeding. Occasional bleeding removed by gauze is required. Bleeding does not threaten lesion site.
GRADE 3	Slight bleeding. Frequent bleeding removed by gauze is required. Bleeding threatens lesion site a few seconds after gauze is removed.
GRADE 4	Moderate bleeding. Frequent bleeding removed by gauze is required. Bleeding threatens lesion site immediately after gauze is removed.
GRADE 5	Severe bleeding. Constant bleeding removed by gauze is required. Bleeding appears faster than can be removed by gauze. Infiltration is not possible.

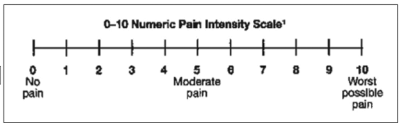

Checklist used for inspection of intralesional infiltration procedures.
